# Efficient Oxidative Polymerization of Hydroxytyrosol Using Horseradish Peroxidase Immobilized on a Magnetic Silica‐Coated Nanocatalyst

**DOI:** 10.1002/gch2.202500064

**Published:** 2025-05-29

**Authors:** Fatih Sevim, Gülşah Aktaş, Hakan Kiziltaş, Fatih Demir, Taner Tekin

**Affiliations:** ^1^ Department of Chemical Engineering Ataturk University Erzurum 25240 Turkey

**Keywords:** enzymatic polymerization, enzyme catalyst, HRP, hydroxytyrosol, immobilization

## Abstract

Enzyme‐based catalysis has gained considerable attention in recent years due to its eco‐friendly and selective nature. However, the inability to recover enzymes after the reaction significantly increases operational costs. In this study, a reusable nanocatalyst Fe_3_O_4_@SiO_2_@APTES@GA@HRP is synthesized and applied in the oxidative polymerization of hydroxytyrosol. Fe_3_O_4_ nanoparticles are prepared using the solvothermal method, followed by silica coating via the Stöber process. Amino‐functionalization is achieved with 3‐aminopropyltriethoxysilane (APTES), and horseradish peroxidase (HRP) is immobilized through glutaraldehyde (GA)‐mediated azomethine bonding. The synthesized catalyst is characterized using SEM, EDS, FTIR, Q‐TOF, ¹H‐NMR, and Zetasizer analyses, all confirming successful immobilization. Polymerization reactions are conducted in acetate buffer (pH 5, 25 °C), resulting in a 73% yield. The catalyst is reusable for up to ten cycles, and the molecular weight of the produced poly(hydroxytyrosol) is approximately 30,000 g mol^−1^. These findings demonstrate the promising application of recyclable enzyme nanocatalysts in green polymer chemistry.

## Graphical abstract

A magnetic Fe₃O₄@SiO₂@APTES@GA nanocatalyst is synthesized for horseradish peroxidase (HRP) immobilization, enabling efficient oxidative polymerization of hydroxytyrosol under mild conditions. The biocatalyst demonstrates high thermal and pH stability, strong reusability over 10 cycles, and effective polymerization performance. Characterizations including SEM, EDS, FTIR, TGA, and kinetic analyses confirmed successful catalyst fabrication and enhanced enzymatic functionality. This work contributes to sustainable green catalysis and nanobiotechnology applications.

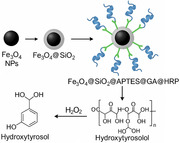

## 1 Introduction

In recent years, the synthesis of biopolymers has emerged as a significant area of research in polymer chemistry. These biopolymers can be synthesized either chemically or enzymatically. Among these approaches, enzymatic polymerization is preferred due to several advantages of enzymes, including high stability, low toxicity, and strong catalytic activity. Enzymes are widely utilized in numerous fields such as medicine, food processing, textiles, chemistry, biochemistry, and various industrial applications.^[^ ^1^, ^2^ ^]^ The growing interest in enzyme‐mediated reactions is largely driven by their compatibility with mild reaction conditions—such as physiological pH, room temperature, and ambient pressure which simplify the polymerization of phenols and vinyl compounds.^[^ ^3^, ^4^ ^]^ Notably, polyphenols synthesized via peroxidase‐catalyzed polymerization exhibit excellent thermal stability.^[^ ^5^ ^]^

The enzymatic polymerization of phenols in aqueous‐organic solvents was first demonstrated by Dordick et al. in 1987, utilizing horseradish peroxidase (HRP) as a catalyst and hydrogen peroxide (H_2_O_2_) as the oxidant.^[^ ^6^ ^]^ Activated HRP can oxidize a broad range of aromatic compounds—including phenols, anilines, phenolic acids, amines, indoles, and sulfonates—producing reactive free radicals that polymerize through carbon–carbon and carbon–oxygen linkages.^[^ ^7^, ^8^ ^]^ However, the instability of free HRP at elevated temperatures and in organic solvents, along with challenges in catalyst recovery, necessitated the development of enzyme immobilization techniques.^[^ ^9^, ^10^, ^11^, ^12^, ^13^, ^14^ ^]^

Enzyme immobilization offers significant advantages, such as enhanced thermal stability, broader operational pH range, and reusability.^[^ ^15^ ^]^ Various immobilization strategies—including non‐covalent adsorption, encapsulation, covalent bonding, and cross‐linking—have been developed to address these challenges.^[^ ^9^ ^]^ Among immobilization supports, magnetic Fe_3_O_4_ nanoparticles have attracted significant attention due to their ease of separation by magnetic fields and potential to improve enzyme thermostability.^[^ ^16^ ^]^ Nevertheless, Fe_3_O_4_ nanoparticles are prone to oxidation under atmospheric conditions, which reduces their stability. Therefore, surfactants, polymers, or noble metals are employed to coat the nanoparticles, enhancing their stability and minimizing water exposure.^[^ ^17^, ^18^ ^]^

Materials such as acrylamide, polysaccharides, chitosan, and silica are commonly used to modify Fe_3_O_4_ surfaces.^[^ ^19^, ^20^ ^]^ Previous studies have investigated the immobilization of enzymes like Thermomyces lanuginosus lipase and HRP on magnetic nanoparticles, achieving notable operational stability.^[^ ^21^ ^]^ For instance, HRP immobilized on Fe_3_O_4_ nanoparticles retained 55% of its initial activity after ten reuses.^[^ ^22^ ^]^ Grebennikova et al. further demonstrated the application of HRP‐immobilized magnetic catalysts in oxidation reactions, including the oxidation of 2,3,6‐trimethylphenol and the development of Fe_3_O_4_APTES/GA/HRP and Fe_3_O_4_/TEOS/APTES/GA/HRP biocatalysts.^[^ ^23^, ^24^ ^]^

Environmental issues associated with olive oil production, particularly olive mill wastewater (OMW), have prompted the search for innovative solutions.^[^ ^25^ ^]^ Phenolic compounds, the primary pollutants in OMW, exceed concentrations of 9.0 g L^−1^ and cause severe soil toxicity.^[^ ^26^, ^27^ ^]^ Hydroxytyrosol (HXT), a major phenolic constituent of OMW, has attracted attention not only for its environmental impact but also for its potent antioxidant properties in biomedical applications.^[^ ^28^ ^]^ Enzymes such as laccase, tyrosinase, and HRP have been utilized to catalyze the polymerization of HXT, with reaction parameters thoroughly studied.^[^ ^29^, ^30^, ^31^, ^32^ ^]^

Immobilization of enzymes onto solid supports has been widely explored to enhance their catalytic performance by improving thermal stability, operational pH tolerance, and reusability. The choice of support material critically affects immobilization efficiency, activity retention, and catalyst robustness. Recent advances have focused on organic–inorganic hybrid supports for superior enzyme functionality. For example, Almulaiky et al. demonstrated that carboxymethylcellulose/ Fe_3_O_4_ hybrids improve the reusability of immobilized peroxidase,^[^ ^33^ ^]^ while El‐Shishtawy et al. reported enhanced catalase stability using chitosan/ZnO/Fe_3_O_4_ composites.^[^ ^34^ ^]^ Amidoximated acrylic fabrics combined with Fe₃O₄ nanoparticles have also been successfully employed for β‐glucosidase immobilization, yielding high operational stability.^[^ ^35^ ^]^ These findings highlight the importance of support selection in the design of efficient and sustainable biocatalysts.

In the present study, magnetic Fe_3_O_4_ nanoparticles were synthesized and subsequently coated with a SiO_2_ layer using the Stöber method to yield Fe_3_O_4_@SiO_2_ nanoparticles. Surface amino functionalization was achieved with 3‐aminopropyltriethoxysilane (APTES). Glutaraldehyde (GA) was then used as a cross‐linking agent to facilitate the covalent immobilization of HRP via azomethine bonding between the enzyme and amino groups. The synthesized Fe_3_O_4_@SiO_2_@APTES@GA@HRP nanocatalyst was subsequently applied to the enzymatic oxidative polymerization of HXT. Characterization of the nanocatalyst was performed using SEM, EDS, and FTIR analyses, while the polymer product was characterized by SEM, EDS, FTIR, Q‐TOF, ¹H‐NMR, and Zetasizer measurements.

## 2 Results and Discussion

**Figure 1:**
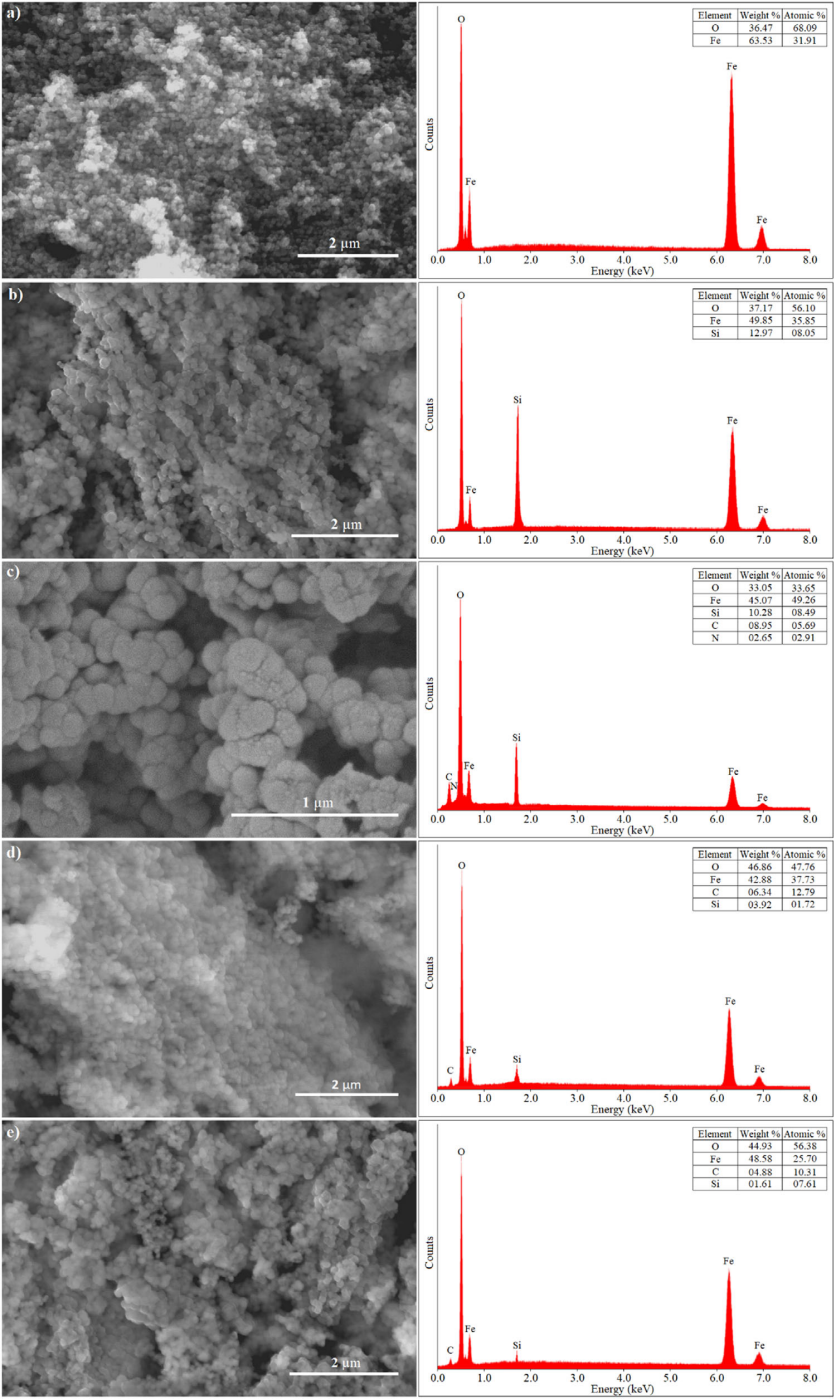
SEM‐EDS images of (a) Fe_3_O_4_, b) Fe_3_O_4_@SiO_2_, c) Fe_3_O_4_@SiO_2_@APTES, d) Fe_3_O_4_@SiO_2_@APTES@GA, and (e) Fe_3_O_4_@SiO_2_@APTES@GA@HRP.

The SEM and EDS characterization results of Fe_3_O_4_, Fe_3_O_4_@SiO_2_, Fe_3_O_4_@SiO_2_@APTES, Fe_3_O_4_@SiO_2_@APTES@GA, and Fe_3_O_4_@SiO_2_@APTES@GA@HRP are shown in **Figure** **1**.

As shown in Figure 1a, the Fe_3_O_4_ nanoparticles exhibit a rough surface and spherical morphology. EDS analysis confirmed the presence of Fe and O elements, characteristic of Fe_3_O_4_.^[^ ^36^ ^]^ In Figure 1b, Fe_3_O_4_@SiO_2_ nanoparticles appear larger with a smoother surface due to the SiO_2_ coating. Granular SiO_2_ nanoparticles are visibly distributed on the Fe_3_O_4_ surface, and EDS analysis confirmed the presence of Fe, O, and Si elements.^[^ ^36^ ^]^

The Fe_3_O_4_@SiO_2_@APTES nanoparticles (Figure 1c) show varying particle sizes and a tendency to aggregate. EDS spectra indicated the successful functionalization with amine groups, as evidenced by the detection of C and N elements in addition to Fe, Si, and O.^[^ ^37^ ^]^ Figure 1d reveals further particle clustering attributed to cross‐linking with GA, and the presence of Fe, Si, O, and C elements was confirmed by EDS analysis.^[^ ^37^ ^]^

In Figure 1e, morphological changes are observed following the immobilization of HRP onto the nanoparticle surface. EDS results indicated the presence of Fe, O, and C elements. Notably, the Fe content increased due to the contribution from the Fe‐containing HRP enzyme, further confirming the successful immobilization process.^[^ ^38^, ^39^ ^]^

**Figure 2:**
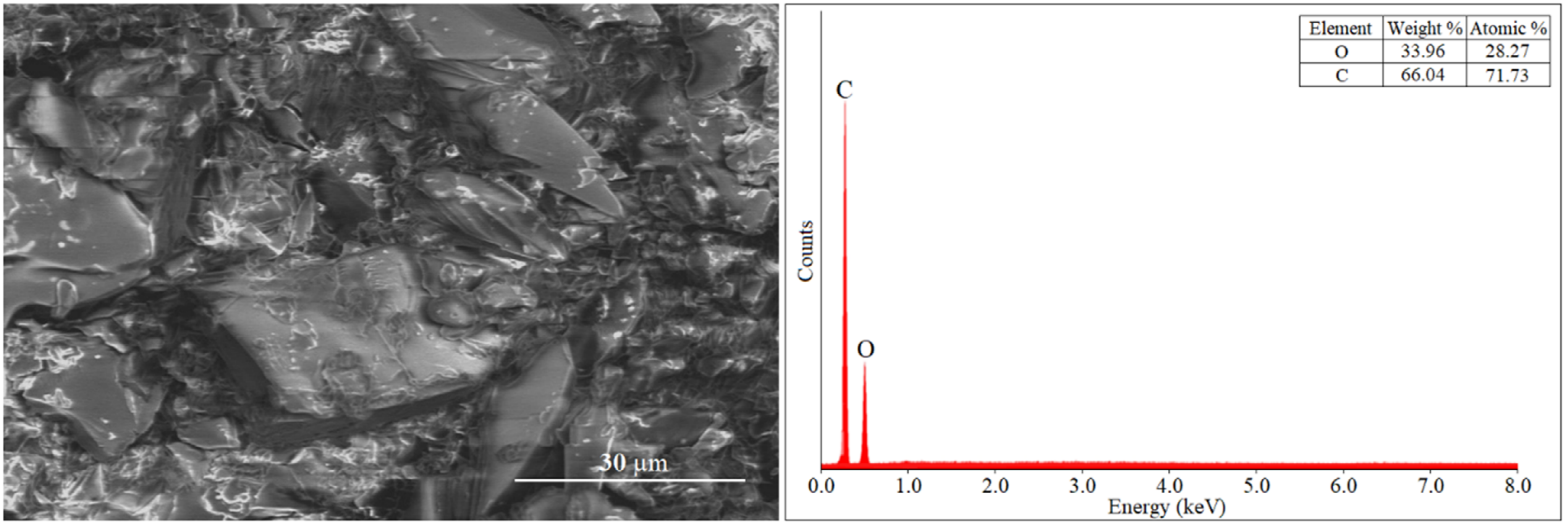
SEM‐EDS images of polymer.

**Figure** **2** displays the SEM image of the synthesized polymer.

EDS analysis confirmed the presence of carbon (C) and nitrogen (N) elements originating from the HXT structure, indicating the successful polymerization. Additionally, the trace amount of sodium (Na) detected is assumed to result from residual buffer salts used during the reaction process.

**Figure 3:**
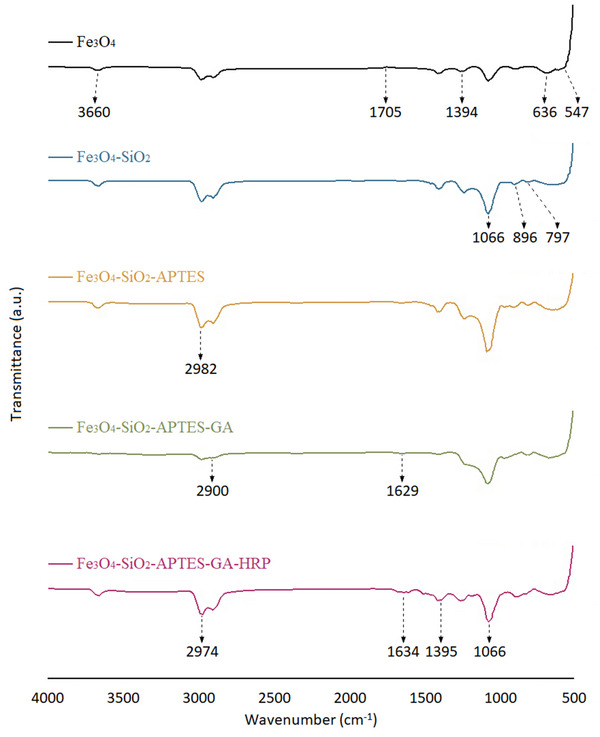
FT‐IR spectra of Fe_3_O_4_, Fe_3_O_4_@SiO_2_, Fe_3_O_4_@SiO_2_@APTES, Fe_3_O_4_@SiO_2_@APTES@GA, and Fe_3_O_4_@SiO_2_@APTES@GA@HRP, illustrating the surface functionalization steps from bare Fe_3_O_4_ nanoparticles to enzyme‐immobilized nanocatalysts.

**Figure 4:**
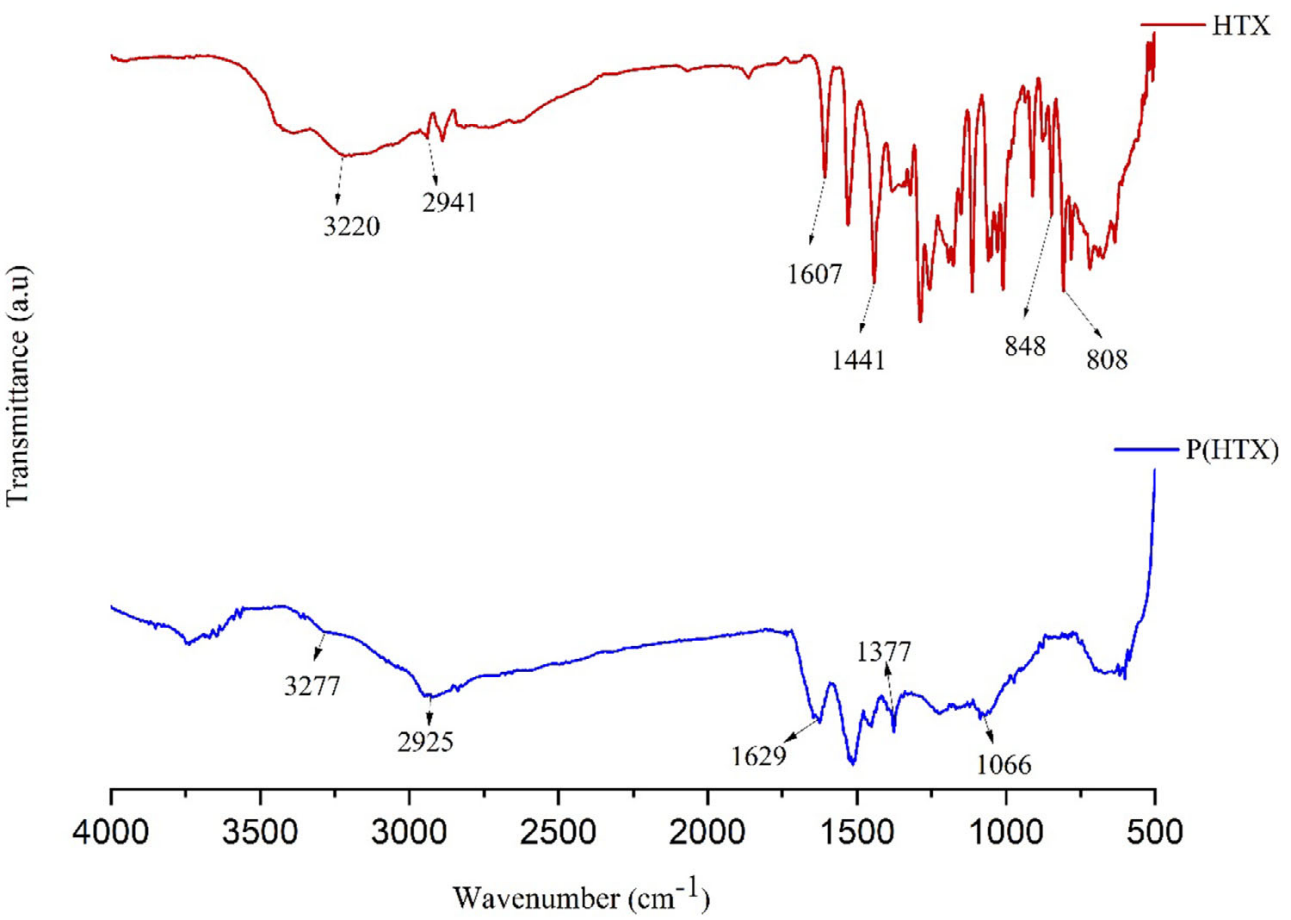
FT‐IR spectra of the HXT monomer and its polymerized form P(HXT), illustrating the structural changes resulting from enzymatic oxidative polymerization.

The FT‐IR spectra of Fe_3_O_4_, Fe_3_O_4_@SiO_2_, Fe_3_O_4_@SiO_2_@APTES, Fe_3_O_4_@SiO_2_@APTES@GA, and Fe_3_O_4_@SiO_2_@APTES@GA@HRP are presented in **Figure** **3**, while the spectra of the monomer and the polymer are displayed in **Figure** **4**.

In the FT‐IR spectrum of Fe_3_O_4_ (Figure 3), the peaks observed at approximately 547 and 636 cm^−1^ correspond to the Fe─O stretching vibrations.^[^ ^40^, ^41^ ^]^ A broad absorption band ≈ 3660 cm^−1^ is attributed to O─H stretching, indicative of surface hydroxyl groups. The bands at 1705 and 1394 cm^−1^ are associated with adsorbed carboxylate groups.^[^ ^42^ ^]^

In the spectrum of Fe_3_O_4_@SiO_2_, the peaks at 1066 and 797 cm^−1^ correspond to the symmetric and asymmetric stretching vibrations of Si─O─Si bonds, respectively.^[^ ^43^ ^]^ Additionally, a peak near 896 cm^−1^ is attributed to free Si─OH groups, confirming the successful coating of SiO_2_ onto the Fe_3_O_4_ nanoparticles.^[^ ^44^, ^45^ ^]^

In the Fe_3_O_4_@SiO_2_@APTES spectrum, the peak at 2982 cm^−1^ is assigned to the symmetric stretching vibrations of C─H groups, indicating successful surface modification with the organosilane linker. In the Fe_3_O_4_@SiO_2_@APTES@GA spectrum, the peak observed at 2900 cm^−1^ corresponds to the C─H stretching vibration associated with GA, while the peak at 1629 cm^−1^ is attributed to the C═O stretching vibration of the aldehyde group.^[^ ^46^ ^]^

In the spectrum of Fe_3_O_4_@SiO_2_@APTES@GA@HRP, the strong band at 1066 cm^−1^ remains, corresponding to Si─O─Si vibrations, consistent with the SiO_2_ structure. A characteristic band at 2974 cm^−1^ is assigned to the stretching vibrations of ─CH_2_ groups. Importantly, the peaks observed at 1634 and 1395 cm^−1^ correspond to the amide I (C═O stretching) and amide II (N─H bending) vibrations, respectively, which are characteristic of ─CONH─ bonds. The presence of these amide bands strongly indicates the successful immobilization of HRP onto the Fe_3_O_4_@SiO_2_@APTES surface.^[^ ^47^, ^48^ ^]^

In the FT‐IR spectrum of the HXT monomer (Figure 4), the peaks at 808 and 848 cm^−1^ are assigned to the C–H bending vibrations of substituted aromatic rings. Peaks observed at 1607 and 1441 cm^−1^ correspond to the C═C stretching vibrations, which are characteristic of the aromatic system of HXT. The band at 2941 cm^−1^ is attributed to the stretching vibrations of aromatic C─H bonds, while the broad bands at 3220 and 3396 cm^−1^ are associated with O─H stretching vibrations.^[^ ^49^, ^50^ ^]^

In the FT‐IR spectrum of the P(HXT), the peak at 1066 cm^−1^ corresponds to the C─O bond stretching, and the band at 1377 cm^−1^ is attributed to the bending vibrations of O─H groups. The peak at 1629 cm^−1^ is assigned to the C═C stretching mode. Additionally, the absorption band at 2925 cm^−1^ corresponds to C─H stretching, and the broad peak observed at 3277 cm^−1^ is due to O─H stretching vibrations.^[^ ^51^ ^]^

**Figure 5:**
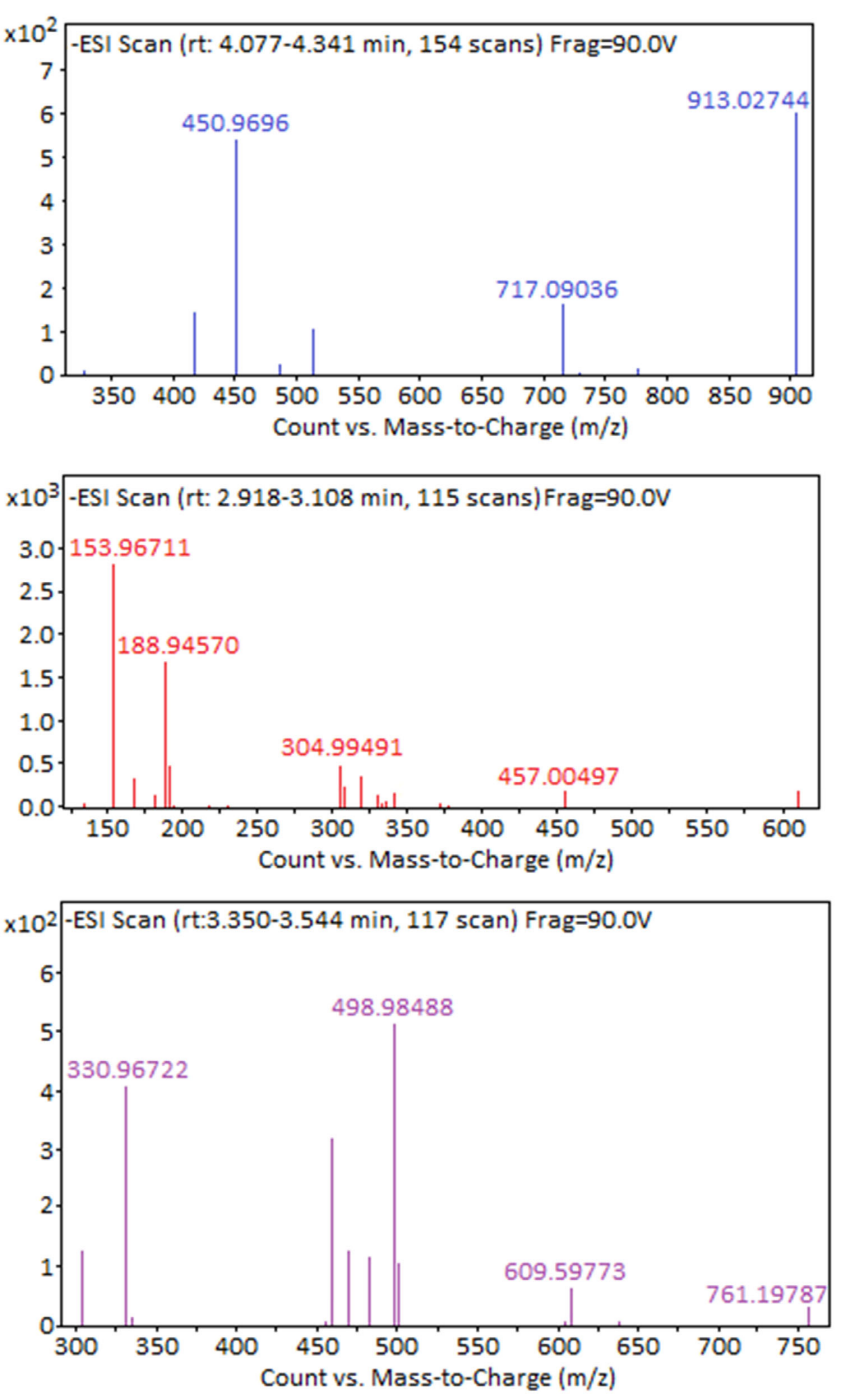
Q‐TOF LC/MS analysis of P(HXT).

The Q‐TOF LC/MS analysis of the polymer is presented in **Figure** **5**.

As shown in Figure 5, the presence of oligomeric products was confirmed in the polymerization catalyzed by the magnetic nanoparticle‐supported HRP (MNP–HRP) catalyst. Peaks detected at m/z values of 153.96711, 304.99491, 457.00497, 609.59773, 761.19787, and 913.02744 correspond to the monomer, dimer, trimer, tetramer, pentamer, and hexamer, respectively.^[^ ^51^ ^]^

In addition, peaks observed at 188.94570, 330.96722, 450.96960, 498.98488, and 717.09036 are attributed to intermediate (precursor) ions formed during the polymerization process. These precursor ions subsequently combine to form the final polymer products (product ions).

**Table** **1** summarizes the measured and theoretical masses, mass errors, identified compounds, and their corresponding molecular formulas.^[^ ^51^, ^52^ ^]^

**Table 1:** Identification of HXT polymer (P(HXT)) compounds by Q‐TOF LC/MS analysis.

No	Measured [m/z]	Formula	Theoretical [m/z]	Error rate [%]	Compounds
1	153.96711	C_8_H_10_O_3_	153.02	0.94	HT monomer
2	304.99491	C_16_H_18_O_6_	305.13	0.14	HT dimer
3	457.00497	C_24_H_26_O_9_	457.16	0.16	HT trimer
4	609.59773	C_32_H_34_O_12_	609.61	0.02	HT tetramer
5	761.19787	C_40_H_42_O_15_	761.25	0.06	HT pentamer
6	913.02744	C_48_H_50_O_18_	913.30	0.28	HT hexamer

**Figure 6:**
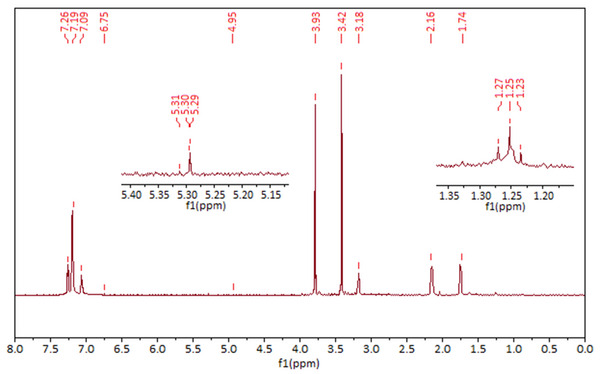
¹H‐NMR spectrum of the P(HXT).

The ¹H‐NMR analysis of the P(HXT), aimed at confirming the structural integrity and successful polymerization, is presented in **Figure** **6**.

In the ¹H‐NMR spectrum shown in Figure 6, the peaks observed at 2.34 and 3.79 ppm correspond to the residual methanol‐d₄ solvent. The aromatic protons (═C─H) of the phenolic ring are identified at chemical shifts of 7.09, 7.19, and 7.26 ppm. Peaks at 1.74 and 2.16 ppm (doublet of triplet) are attributed to the methylene protons (─CH_2_) linked to the aromatic ring. The signal at 3.18 ppm corresponds to ─CH_2_ attached to hydroxyl groups. A distinct peak at 5.30 ppm is assigned to the proton of the hydroxyl (─OH) group connected to the methylene unit; however, the hydroxyl protons on the aromatic ring are not observed, likely due to rapid proton exchange in deuterated solvent.

In the polymerized product, very low‐intensity signals were observed, attributed to the minimal residues of the magnetic catalyst after removal.^[^ ^51^, ^52^ ^]^ It is also known that the reaction pH affects the polymerization mechanism: at acidic pH, C─O linkages are predominantly formed, whereas basic pH conditions favor the formation of C─C bonds between monomer units.^[^ ^53^ ^]^

The apparent molecular weight (Mw) of the polymer was estimated to be ≈30,000 g mol^−1^ based on hydrodynamic size measurements performed using a Zetasizer Nano ZSP instrument.

The solubility properties of the polymer were evaluated in various solvents, including pure water, acetone, methanol, toluene, chloroform, THF, DMSO, and DMF. The polymer was observed to be soluble in pure water and DMF, partially soluble in methanol and THF, and insoluble in acetone, toluene, chloroform, and DMSO.

To provide a more quantitative assessment, the solubility of the polymer was determined by preparing saturated solutions in various solvents, followed by filtration and drying to calculate the maximum solubility values (mg/mL). Additionally, the pH‐dependent solubility behavior was evaluated using phosphate buffer solutions across a pH range from 3 to 9. The corresponding results are summarized in **Table** **2**.

**Table 2:** Solubility and pH Sensitivity of the P(HXT).

Solvent / pH Condition	Solubility [mg/mL]	Observation
Water (pH 7)	2.5	Slightly soluble
Ethanol	4.8	Soluble
Methanol	5.2	Soluble
DMSO	9.6	Fully soluble
PBS (pH 7.4)	3.2	Moderately soluble
pH 3 Buffer	1.5	Partially precipitated
pH 5 Buffer	2.3	Slight turbidity
pH 7 Buffer	3.0	Clear solution
pH 9 Buffer	4.5	Increased solubility

The polymer exhibited the highest solubility in DMSO (9.6 mg mL^−1^), followed by methanol (5.2 mg mL^−1^) and ethanol (4.8 mg mL^−1^). In aqueous environments, the solubility was moderate (2.5–3.2 mg mL^−1^ under neutral pH). Under alkaline conditions (pH 9), solubility increased to 4.5 mg mL^−1^, whereas under acidic conditions (pH 3), solubility decreased below 2 mg mL^−1^, indicating pH‐responsive behavior of the polymer.

**Figure 7:**
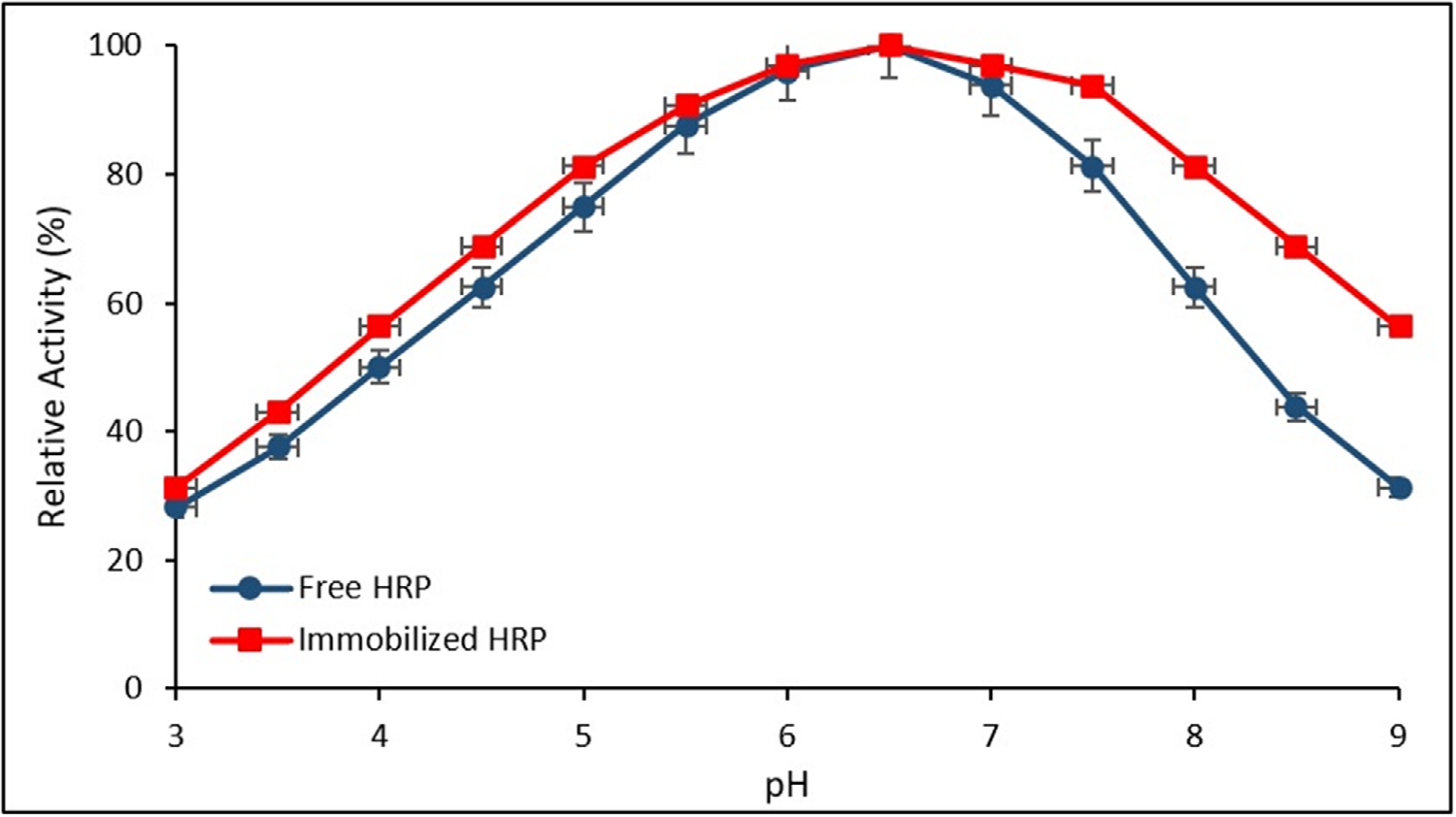
Effect of pH on the relative activities of free and immobilized HRP (*Data are presented as mean ± SD (n = 3, p < 0.05))*.

**Figure** **7** illustrates the relative activities of free and immobilized HRP over a pH range of 3.0–9.0. Error bars represent the SD from three independent experiments (*n* = 3).

Both enzyme forms exhibited maximum catalytic activity within the pH range of 6.5–7.0. Notably, the immobilized HRP retained higher relative activity compared to its free counterpart under both acidic and alkaline conditions, suggesting enhanced pH stability as a result of immobilization.

These results align well with previous studies, which have demonstrated that enzyme immobilization improves stability under sub‐optimal pH conditions. The broader operational pH range observed for immobilized HRP is likely attributable to the protective microenvironment provided by the Fe_3_O_4_@SiO_2_@APTES support matrix. Similar observations were reported by Chang et al. (2014) and El‐Shishtawy et al. (2021), who found that functionalized magnetic nanoparticle supports significantly enhance the structural integrity and catalytic resilience of immobilized HRP.^[^ ^34^, ^54^ ^]^

**Figure 8:**
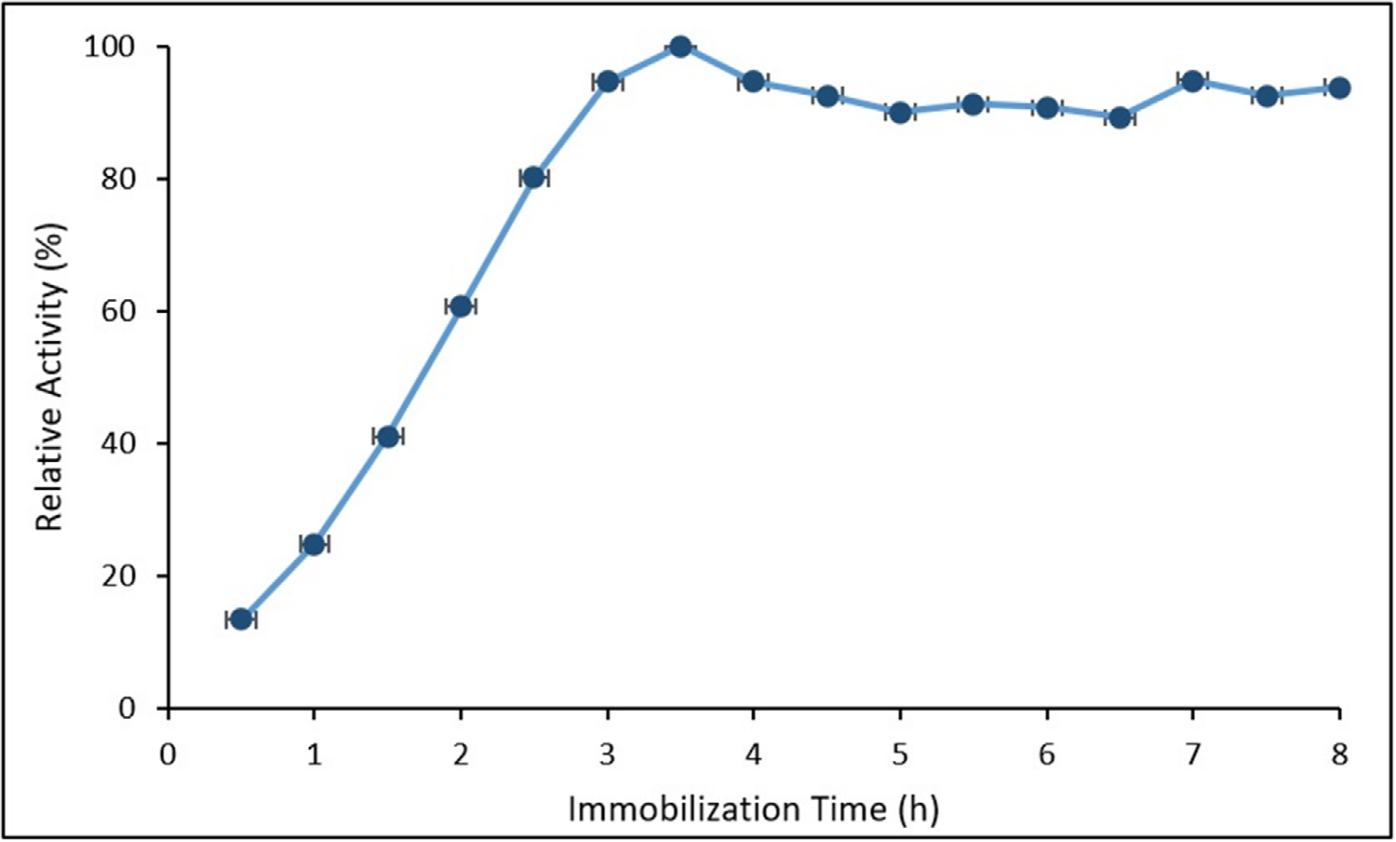
Effect of immobilization time on the relative activity of HRP immobilized on Fe_3_O_4_@SiO_2_@APTES (*Data are presented as mean ± SD (n = 3, p < 0.05))*.

The effect of immobilization time on HRP activity is presented in **Figure** **8**. Error bars represent the SD from three independent experiments (*n* = 3).

A progressive increase in relative activity was observed between 0.5 and 3.5 h, reaching a maximum at 3.5 h. Beyond this time, the activity plateaued, exhibiting slight fluctuations between 90% and 95%. The initial rise corresponds to the gradual binding of HRP molecules onto the support surface, while the stabilization phase is likely associated with surface saturation and steric hindrance, limiting further enzyme attachment. These observations are consistent with previous studies by El‐Shishtawy et al. (2021) and Chang et al. (2014), which reported optimal immobilization times of ≈3–4 h for HRP systems using magnetic nanoparticle supports.^[^ ^34^, ^54^ ^]^

**Figure 9:**
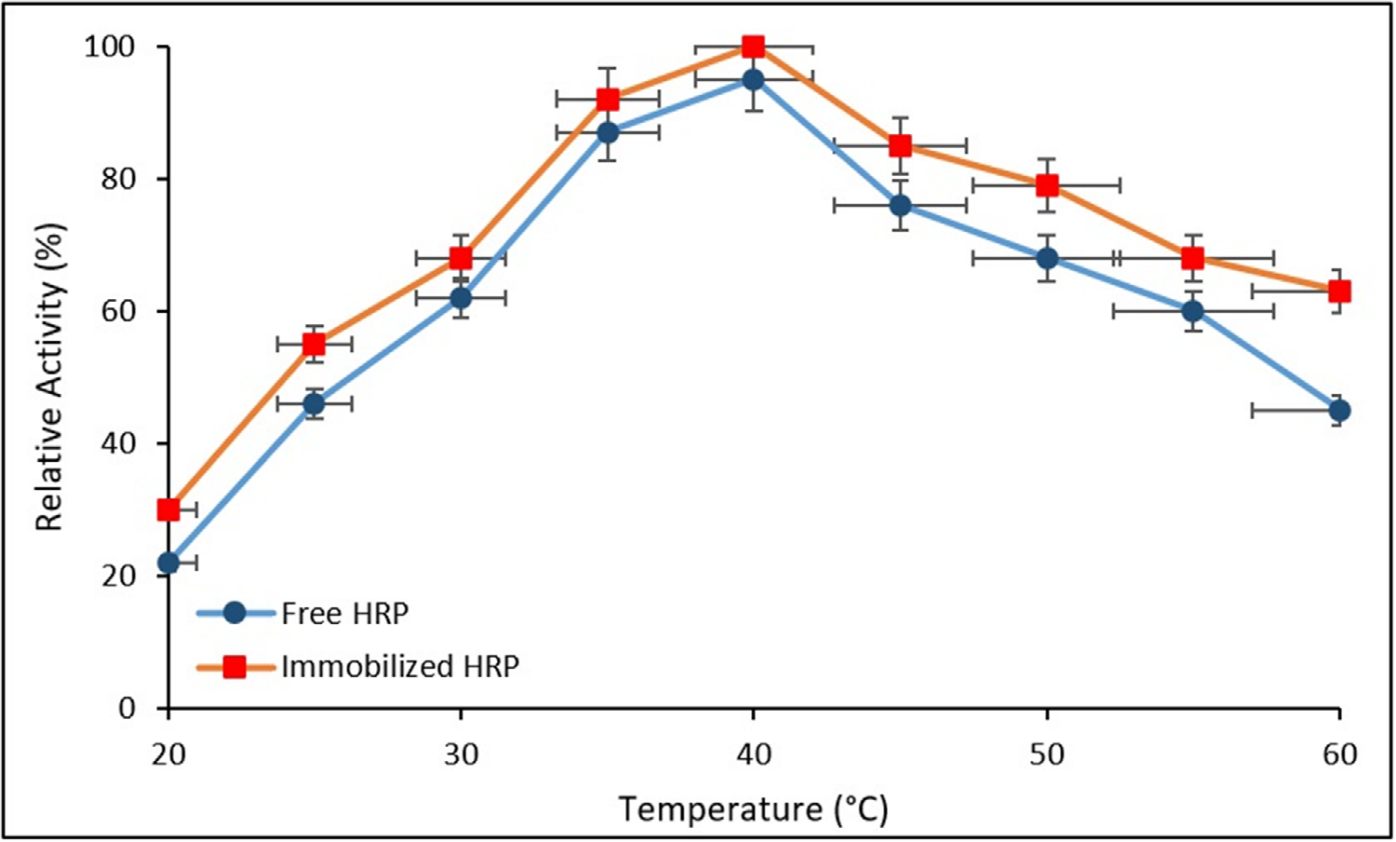
Effect of temperature on the relative activity of free and immobilized HRP (*Data are presented as mean ± SD (n = 3, p < 0.05))*.

The effect of temperature on the relative activity of free and immobilized HRP is discussed in **Figure** **9**. Error bars represent the SD from three independent experiments (*n* = 3).

As shown in Figure 9, both free and immobilized HRP exhibited maximum catalytic activity at 40 °C. However, the immobilized enzyme retained significantly higher relative activity at elevated temperatures (45–60 °C), whereas the free enzyme displayed a sharp decline beyond 40 °C. This indicates that immobilization conferred enhanced thermal stability, likely due to the restricted conformational flexibility and the stabilizing microenvironment provided by the Fe_3_O_4_@SiO_2_@APTES support matrix. These findings are consistent with previous reports demonstrating improved thermal tolerance in immobilized peroxidases.^[^ ^34^ ^]^

**Figure 10:**
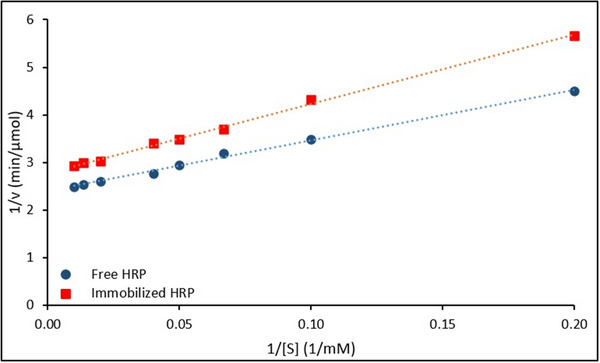
Lineweaver–Burk plots of free and immobilized HRP.

The Lineweaver–Burk plots comparing the kinetic behavior of free and immobilized HRP are presented in **Figure** **10**.

The kinetic behavior of free and immobilized HRP was evaluated using Lineweaver–Burk plots (Figure 10), and the corresponding linear regression equations and kinetic parameters are summarized in **Table** **3**.

**Table 3:** Kinetic parameters for HXT polymerization catalyzed by free and immobilized HRP.

Enzymes	Lineweaver–Burk equation	R^2^	V_max_ [µmol/min]	K_m_ [mM]
Free HRP	$\;\frac{1}{v}=10.661\frac{1}{\left[S\right]}+2.397$	0.987	0.42	4.45
Immobilized HRP	$\;\frac{1}{v}=14.556\frac{1}{\left[S\right]}+2.780$	0.981	0.36	5.24

Analysis of the plots revealed that immobilization resulted in a decrease in the maximum reaction rate (V_max_) and an increase in the Michaelis–Menten constant (K_m_). Specifically, the V_max_ and K_m_ values for the free enzyme were calculated as 0.42 µmol min^−1^ and 4.45 mM, respectively, while for the immobilized enzyme, the corresponding values were 0.36 µmol min^−1^ and 5.24 mM.

The observed decrease in V_max_ is attributed to reduced conformational flexibility or diffusion limitations within the support matrix, whereas the increase in K_m_ suggests a reduced substrate affinity, likely due to steric hindrance or partial obstruction of the enzyme active sites.

These results are consistent with previous findings.^[^ ^34^, ^54^, ^55^ ^]^ For instance, Almulaiky et al. (2019) reported a decrease in V_max_ (from 0.380 to 0.331 U mL^−1^) and an increase in K_m_ (from 4.81 to 5.71 mM) upon immobilizing HRP on Fe₃O₄@chitosan. Similarly, Chang et al. (2014) observed increased K_m_ and reduced V_max_ values when HRP was immobilized on NH₂‐functionalized Fe₃O₄/SiO₂ nanoparticles.^[^ ^33^, ^47^ ^]^

Thus, the kinetic shifts observed in this study further validate the successful immobilization of HRP onto the magnetic nanocarrier system.

**Figure 11:**
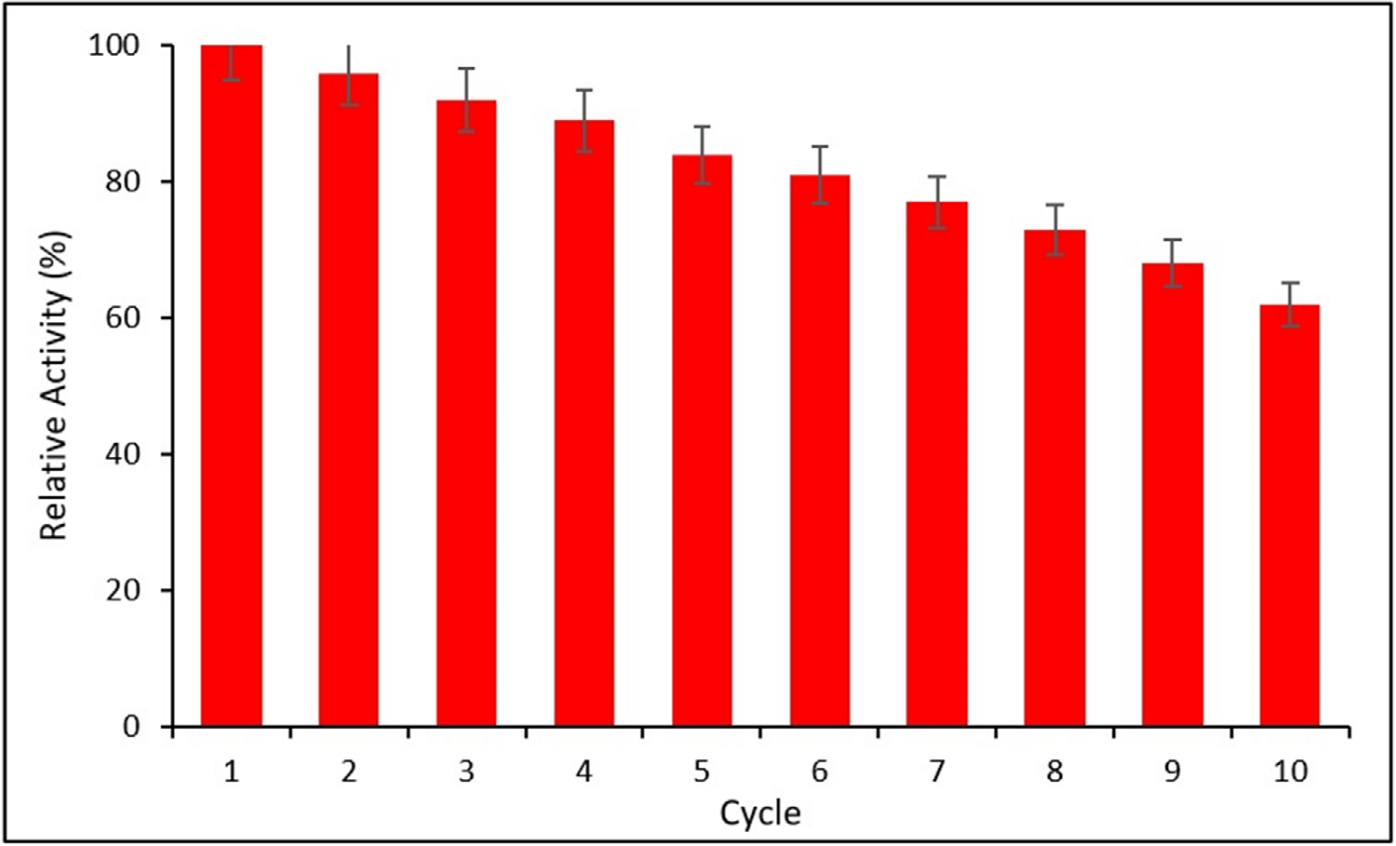
Reusability performance of immobilized HRP over 10 consecutive catalytic cycles (*Data are presented as mean ± SD (n = 3, p < 0.05))*.

The reusability performance of the immobilized HRP was evaluated over 10 successive polymerization cycles. As shown in **Figure** **11**, the enzyme retained 62% of its initial activity after 10 cycles, demonstrating good operational stability. The gradual decline in activity is likely due to enzyme leaching or partial structural degradation, consistent with previous reports.^[^ ^34^, ^47^ ^]^ Error bars represent the SD from three independent experiments (*n* = 3).

**Figure 12:**
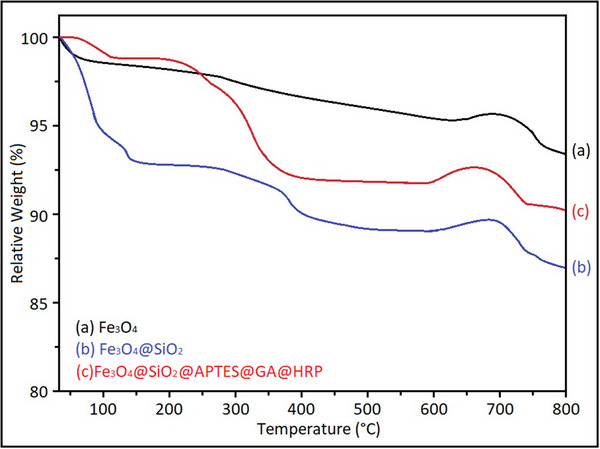
TGA results of (a) Fe_3_O_4_, (b) Fe_3_O_4_@SiO_2_, and (c) Fe_3_O_4_@SiO_2_@APTES@GA@HRP.

The TGA was additionally performed to confirm the successful immobilization of HRP on the magnetic support, with the corresponding results presented in **Figure** **12**.

The pristine Fe_3_O_4_ nanoparticles exhibited minimal weight loss (≈2.3%), reflecting their high intrinsic thermal stability. Upon silica coating, the Fe_3_O_4_@SiO_2_ nanocomposite demonstrated a moderate weight loss (≈6.0%), which is primarily attributed to the elimination of surface hydroxyl groups and residual organic species.

In contrast, the Fe_3_O_4_@SiO_2_@HRP sample showed a significant overall weight loss (≈14.6%) over the temperature range of 30–600 °C. Two distinct degradation stages were observed: the first major step occurred between 150–400 °C (7.8%), and the second step appeared between 400–600 °C (4.2%), both indicative of the thermal decomposition of the immobilized HRP enzyme. After correcting for the inorganic residue, the net enzyme loading was estimated to be ≈10–12 wt.%.

These findings are consistent with previous reports on HRP immobilization systems. For example, El‐Shishtawy et al. (2021) reported an enzyme‐related weight loss of ≈9.8% in ZnO/chitosan‐based HRP composites, as determined by thermogravimetric analysis.^[^ ^34^ ^]^ The slightly higher enzyme loading observed in the present study (≈10–12%) suggests that the APTES‐functionalized Fe_3_O_4_@SiO_2_ surface offered a highly efficient and stable platform for enzyme immobilization.^[^ ^56^ ^]^

## 3 Conclusion

In this study, a reusable magnetic nanocatalyst (Fe_3_O_4_@SiO_2_@APTES@GA@HRP) was successfully synthesized and applied in the oxidative polymerization of HXT under mild reaction conditions. The Fe_3_O_4_ core was synthesized via the solvothermal method and coated with SiO_2_ using the Stöber process, followed by amino‐functionalization and covalent immobilization of HRP through GA cross‐linking.

Comprehensive characterization of the synthesized catalyst and the resulting polymer was conducted using SEM, EDS, FTIR, ¹H‐NMR, Q‐TOF LC/MS, Zetasizer, and TGA analyses. SEM‐EDS results confirmed the morphological integrity and elemental distribution at each functionalization step, while FTIR spectra revealed the successful modification and immobilization of functional groups. Q‐TOF LC/MS analysis identified the formation of HXT oligomers (dimer, trimer, up to hexamer), while ¹H‐NMR spectroscopy confirmed the polymer structure. The polymer exhibited an estimated molecular weight of ≈30,000 g mol^−1^.

The catalytic performance of free and immobilized HRP was systematically evaluated under varying pH, temperature, and immobilization conditions. The immobilized HRP demonstrated enhanced stability across a broader pH (3.0–9.0) and temperature (20–60 °C) range compared to free HRP. Kinetic analysis revealed a slight decrease in V_max_ (0.42 to 0.36 µmol min^−1^) and an increase in K_m_ (4.45 to 5.24 mM) upon immobilization, attributed to diffusional limitations and steric effects, which is consistent with previous studies.

The operational reusability of the immobilized enzyme was confirmed over 10 consecutive catalytic cycles, with the retained activity reaching 62% after the tenth reuse, demonstrating good durability. Thermogravimetric analysis indicated an enzyme loading of ≈10–12 wt.%, further supporting successful immobilization.

Additionally, the solubility behavior of the produced polymer was assessed, revealing good solubility in water and polar solvents, and a pH‐dependent solubility profile, making it a promising candidate for stimuli‐responsive applications.

Overall, the synthesized Fe_3_O_4_@SiO_2_@APTES@GA@HRP nanocatalyst represents an efficient, recyclable, and robust platform for green polymerization processes. These findings provide valuable insights into the design of advanced biocatalytic systems for sustainable polymer synthesis and offer potential applications in environmental and biomedical fields.

## 4 Experimental Section

### Materials

The chemicals used throughout the synthesis processes included: iron(III) chloride hexahydrate (FeCl_3_·6H_2_O, Sigma‐Aldrich, 98%), ethylene glycol (EG, C_2_H_6_O_2_, Sigma‐Aldrich, 98%), sodium acetate (NaAc, CH_3_COONa, Sigma‐Aldrich, 99%), polyethylene glycol (PEG, C_2n_H_4n+2_O_n+1,_ Sigma‐Aldrich, M_r_:7000–9000), ethanol (C_2_H_5_OH Isolab, 99.9%), ammonium hydroxide (NH_4_OH, Sigma‐Aldrich, 25%), tetraethyl orthosilicate (TEOS, Si(OC_2_H_5_)_4_, Sigma‐Aldrich, 99%), 3‐aminopropyltriethoxysilane (APTES, C_9_H_23_NO_3_Si, Sigma‐Aldrich, 99%), monosodium phosphate (NaH_2_PO_4_, BLD Pharmatech, 99%), disodium hydrogen phosphate dihydrate (Na_2_HPO_4_·2H_2_O, Isolab, 99.5%), sodium hydroxide (NaOH, Isolab, 99%), hydrochloric acid (HCI, Merck, 37%), horseradish peroxidase (HRP, Sigma‐Aldrich), 3‐hydroxytyrosol (HXT, C_8_H_10_O_3_, BLD Pharmatech), hydrogen peroxide (H_2_O_2_, Tekkim, 35%), methanol (CH_3_OH, Merck, 99.7%), dimethylformamide (DMF, C_3_H_7_NO, Tekkim, 99%), acetone (C_3_H_6_O, Sigma‐Aldrich, 99.5%), toluene (C_7_H_8_, Sigma‐Aldrich, 99.8%), chloroform (CHCl_3_, Sigma‐Aldrich, ≥99%), dimethyl sulfoxide (DMSO, C_2_H_6_OS, Sigma‐Aldrich, ≥99.9%), tetrahydrofuran (THF, C_4_H_8_O, Sigma‐Aldrich, ≥99.9%), and glutaraldehyde (GA, C_5_H_8_O_2_, Tekkim, 50%).

### Fe_3_O_4_ Synthesis

Fe_3_O_4_ nanoparticles were synthesized via a solvothermal method. Briefly, 5.6 g of FeCl_3_.6H_2_O was dissolved in 80 mL of EG under ultrasonication until a homogeneous light‐yellow solution was obtained. Subsequently, 4 g of PEG and 14.4 g of NaAc were added to the solution, followed by magnetic stirring for 15 min. The resulting mixture was transferred into a 200 mL Teflon‐lined stainless‐steel autoclave and heated at 200 °C for 12 h under static conditions. After cooling to room temperature, the black Fe_3_O_4_ precipitates were collected using an external magnet. The isolated nanoparticles were washed five times with deionized water and ethanol sequentially and then dried in an oven at 60 °C for 3 h.^[^ ^57^ ^]^

### Fe_3_O_4_@SiO_2_ Synthesis

Fe_3_O_4_@SiO_2_ nanoparticles were synthesized using the Stöber method. Initially, 0.1 g of Fe_3_O_4_ from the previous step was added to a mixture of 80 mL of ethyl alcohol and 20 mL of deionized water, and sonicated for 15 min. Subsequently, 5 mL of 25% (w/w) NH_4_OH was introduced into the homogeneous solution, followed by an additional 5 min of mixing. Then, 1 mL of TEOS was gradually added dropwise. The solution was then stirred at 45% amplitude for 3 h. After completion, the Fe_3_O_4_@SiO_2_ particles were magnetically separated from the solution. The particles were washed five times with distilled water and ethanol, and then dried in an oven at 60 °C for 3 h. Finally, the dried nanoparticles were calcined at 200 °C for 2 h.^[^ ^58^ ^]^

### Fe_3_O_4_@SiO_2_@APTES Synthesis

Fe_3_O_4_@SiO_2_ nanoparticles (0.50 g) were dispersed in a mixed solvent containing 100 mL of ethanol and 100 mL of deionized water. The dispersion was sonicated at room temperature for 30 min to achieve uniform suspension. Subsequently, 4 mL of APTES was added dropwise under continuous sonication. The mixture was further sonicated for an additional 1 h to facilitate the functionalization process. The resulting Fe_3_O_4_@SiO_2_@APTES nanoparticles were collected using an external magnet, washed five times sequentially with deionized water and ethanol, and dried in an oven at 75 °C for 6 h.

### Fe_3_O_4_@SiO_2_@APTES@GA Synthesis

A phosphate buffer solution (PBS) was prepared by dissolving 0.35 g of disodium hydrogen phosphate dihydrate and 0.48 g of monosodium phosphate in 50 mL of deionized water. The solution was briefly sonicated to ensure complete dissolution. The pH was adjusted to the desired value (7.8) using 0.1 M NaOH or 0.1 M HCl, as required, and confirmed with a calibrated pH meter.

Subsequently, 0.45 g of Fe_3_O_4_@SiO_2_@APTES nanoparticles was added to 40 mL of the prepared PBS solution (pH 7.8) and sonicated for 1 h at room temperature to achieve uniform dispersion. After sonication, the mixture was transferred to a magnetic stirrer, and 3.5 mL of 50% GA solution (pre‐adjusted to pH 7.0) was added dropwise using a Pasteur pipette. The reaction mixture was stirred continuously at 150 rpm at room temperature (25 °C) for 24 h to facilitate the activation of amino groups on the nanoparticle surface.

### Fe_3_O_4_@SiO_2_@APTES@GA Enzyme Immobilization

25 mg of HRP was dissolved in 50 mL of PBS (pH 7.0) and sonicated at room temperature for 30 min to ensure complete dispersion. Subsequently, 0.40 g of Fe_3_O_4_@SiO_2_@APTES@GA nanoparticles was added to the HRP solution. The resulting mixture was transferred into a clean glass container and stirred continuously at 150 rpm for 48 h at room temperature. After incubation, the Fe_3_O_4_@SiO_2_@APTES@HRP nanocatalysts were collected using an external magnet, washed several times with deionized water to remove unbound enzyme, and dried for further use.

### Polymerization of HXT with Magnetic Nanocatalyst

First, the acetate buffer needed for the polymerization reaction was prepared. To prepare the acetate buffer, a mixture of 0.2 m 82 mL acetic acid and 0.2 m 18 mL sodium acetate solutions was diluted to 200 mL with pure water. The pH was adjusted to 5 using 0.1 m NaOH to increase the pH and 0.1 m HCl to decrease it, continuing until the desired pH was reached.

Next, 10 mL of pure water, 10 mL of pH 5 acetate buffer, and 100 mg of HXT were added to 80 mg of the magnetic nanocatalyst to initiate the polymerization reaction. As an oxidizer, 500 µl of 99.8% pure H_2_O_2_ was added to the solution using a micropipette every 15 min, for a total of 14 additions. A color change to red‐brown was observed after the first addition of H_2_O_2_. The solution was stirred in an evaporator at 25 °C and 180 rpm for 24 h. After this period, the polymer products were separated from the catalyst using magnetization. The separated product was washed several times with pure water, dried, and stored at ‐20 °C for analysis. The magnetic material was washed several times, first with buffer and then with pure water, and then stored in pure water at 4 °C for reuse.

### Characterization

The morphology, size, and elemental composition of the nanoparticles and polymer were characterized using scanning electron microscopy (SEM, Zeiss Sigma 300) combined with energy‐dispersive X‐ray spectroscopy (EDS). Surface functional groups were analyzed by Fourier transform infrared spectroscopy (FTIR, Vertex 80v) in transmission mode over the frequency range of 4000–500 cm^−1^. The structure of the polymer product was confirmed by ^1^H‐NMR spectroscopy (Bruker 400 MHz) using methanol‐D_4_ as the solvent and tetramethylsilane as the internal standard. The particle size distribution and surface charge (zeta potential) were determined using dynamic light scattering (DLS) and electrophoretic light scattering measurements performed with a Malvern Zetasizer Nano ZSP. Although the Zetasizer primarily measures the hydrodynamic diameter of particles, the molecular weight estimation reported in this study was derived based on assumed polymer density correlations. Thermal stability and enzymatic immobilization were investigated by thermogravimetric analysis (TGA) using a PerkinElmer Pyris system under nitrogen atmosphere, with a heating rate of 10 °C min^−1^ from 30 °C to 800 °C. Additionally, the polymer's molecular characteristics were examined via quadrupole time‐of‐flight mass spectrometry (Q‐TOF, Bruker Impact II).

### Kinetic Evaluation of Free and Immobilized HRP

The enzymatic behavior of free and immobilized HRP was systematically evaluated under different conditions.

First, the effect of pH on enzymatic activity was examined. Reactions were conducted in 50 mm buffer solutions with pH values ranging from 3.0 to 9.0. Relative activities were determined by measuring absorbance changes at 280 nm, and the maximum activity was taken as 100%.

Next, the effect of temperature on catalytic activity was studied by performing enzymatic reactions at various temperatures ranging from 20 to 70 °C under otherwise identical conditions. The relative activities at each temperature were recorded and compared.

To optimize the immobilization parameters, the effect of immobilization time was evaluated by varying the incubation time between the Fe_3_O_4_@SiO_2_@APTES nanoparticles and the HRP solution from 0.5 to 10 h. Similarly, the impact of immobilization temperature was assessed by conducting immobilization at temperatures between 4 °C and 45 °C, and measuring the resulting catalytic activities.

The operational stability and reusability of the immobilized enzyme were tested by performing consecutive reaction cycles. After each reaction cycle, the nanocatalyst was magnetically separated, washed, and reused in a fresh reaction mixture. The relative activities after each reuse were determined.

Finally, the kinetic parameters of the enzymatic reactions were determined. HXT solutions with varying concentrations (0.5–5.0 mm) were used as substrates in the presence of 1 mm H_2_O_2_ at pH 7.0 and 25 °C. The initial reaction rates (v₀) were calculated based on absorbance measurements at 280 nm. The data were analyzed using the Michaelis–Menten model:

(1) $$v=\frac{V_{max}x\left[S\right]}{K_{m}+\left[S\right]}\;$$

where v is the initial reaction rate (µmol/min), [S] is the substrate concentration (mM), V_max_​ is the maximum reaction rate, and K_m_​ is the Michaelis–Menten constant.

The kinetic parameters were further determined using the Lineweaver–Burk linearization method, described by the following Equation (2):

(2) $$\frac{1}{v}=\frac{K_{m}}{V_{max}}\;x\frac{1}{\left[S\right]}+\frac{1}{V_{max}}$$

Plots of 1/v versus 1/[S] were generated to calculate V_max_ and K_m_ values for both free and immobilized HRP.

### Statistical Analysis

All experiments were conducted in triplicate (*n* = 3) unless otherwise stated. Data are presented as mean ± standard deviation (SD). No pre‐processing (such as normalization or transformation) or removal of outliers was performed. Statistical analysis was carried out using OriginPro 2023 software (OriginLab Corporation, USA). Differences between groups were assessed using one‐way analysis of variance (ANOVA) followed by Tukey's post‐hoc test for multiple comparisons. A p‐value of less than 0.05 was considered statistically significant.

## Conflict of Interest

The authors declare no conflict of interest.

## Data Availability

The data that support the findings of this study are available from the corresponding author upon reasonable request.
